# Supplementary material for: Long‐Read Sequencing Reveals Extensive DNA Methylations in Human Gut Phagenome Contributed by Prevalently Phage‐Encoded Methyltransferases
Source: Adv Sci (Weinh). 2023 Jun 29;10(25):2302159. doi: 10.1002/advs.202302159 (PMC10477858; doi:10.1002/advs.202302159)
Supplement: Supplementary file 1 — Supporting Information [file ADVS-10-2302159-s003.pdf]

ADVANCED SCIENCE
Open Access

Supporting Information

for *Adv. Sci.*, DOI 10.1002/advs.202302159

Long-Read Sequencing Reveals Extensive DNA Methylations in Human Gut Phagenome Contributed by Prevalently Phage-Encoded Methyltransferases

Chuqing Sun, Jingchao Chen, Menglu Jin, Xueyang Zhao, Yun Li, Yanqi Dong, Na Gao, Zhi Liu, Peer Bork*, Xing-Ming Zhao* and Wei-Hua Chen**

Supporting Tables

Table S1. A list of 8848 phages and related information in details, see excel file Table S1.
Table S2. MTases identified from the 8848 phages, UHGG2 genomes, and their clustering results, excel file Table S2.

Supporting Figures

Figure S1

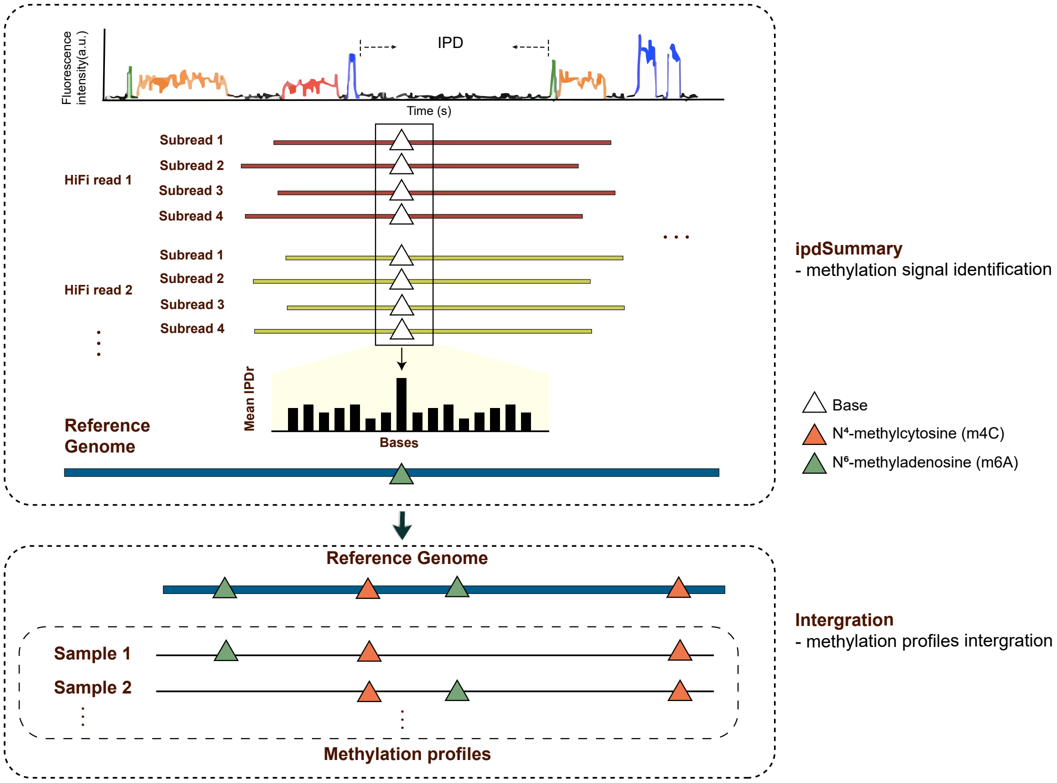

Figure S1, DNA methylation identification using SMRT sequencing.

Figure S2

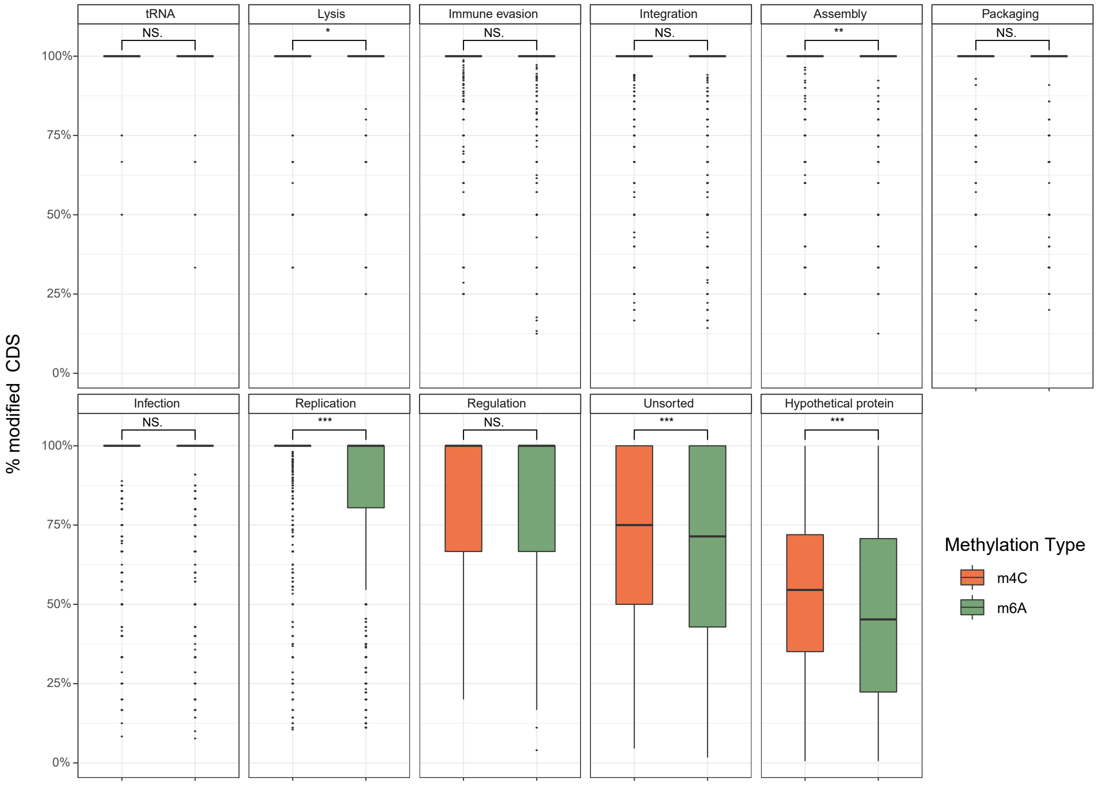

Figure S2, Differential distribution patterns of m6A and m4C modifications in coding genes with different functions.

Figure S3

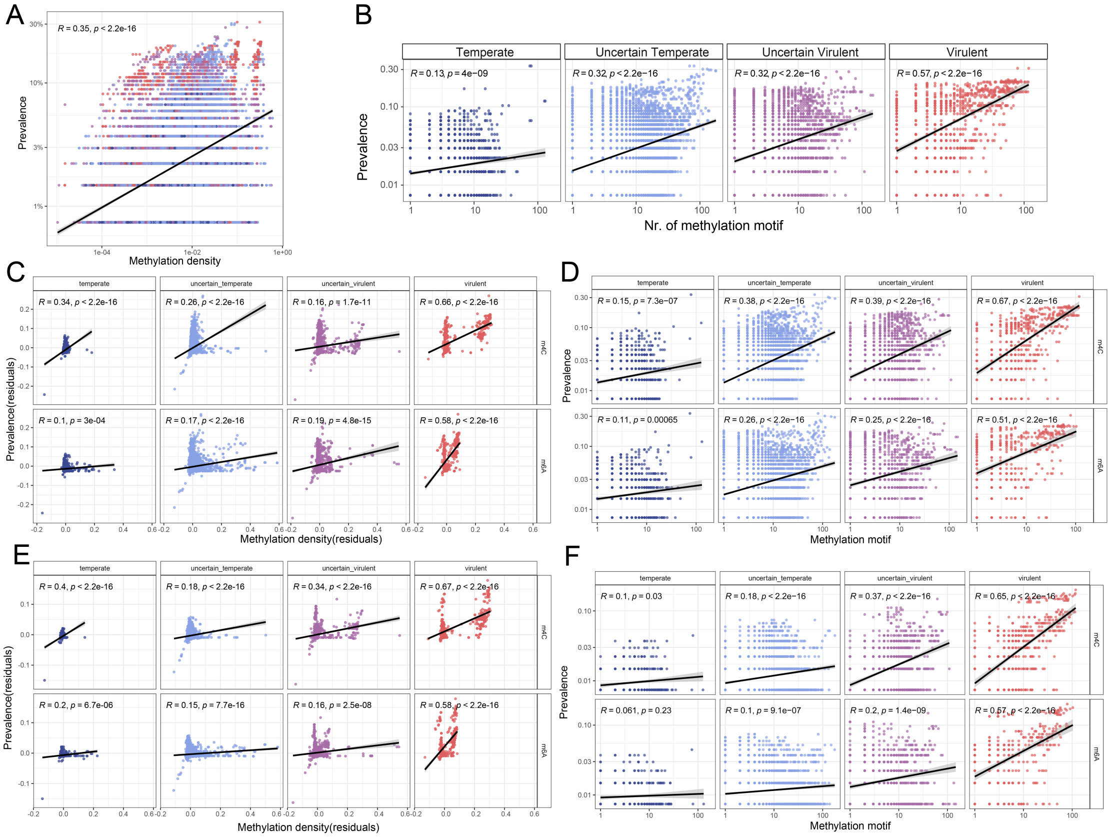

Figure S3, The fitness of the CHGV-HQ phages, measured by the prevalence (lower panels) across 104 fecal samples, was positively correlated with **A)** overall DNA methylation density, **B)** the total numbers of methylation motifs, **C)** overall DNA methylation density of m4C and m6A and **D)** numbers of methylation motifs of m4C and m6A. **E,F)** The prevalence was calculated by using an abundance cutoff of 5 as the presence/absence threshold, showing that changing the abundance cutoff did not affect our main results

Figure S4

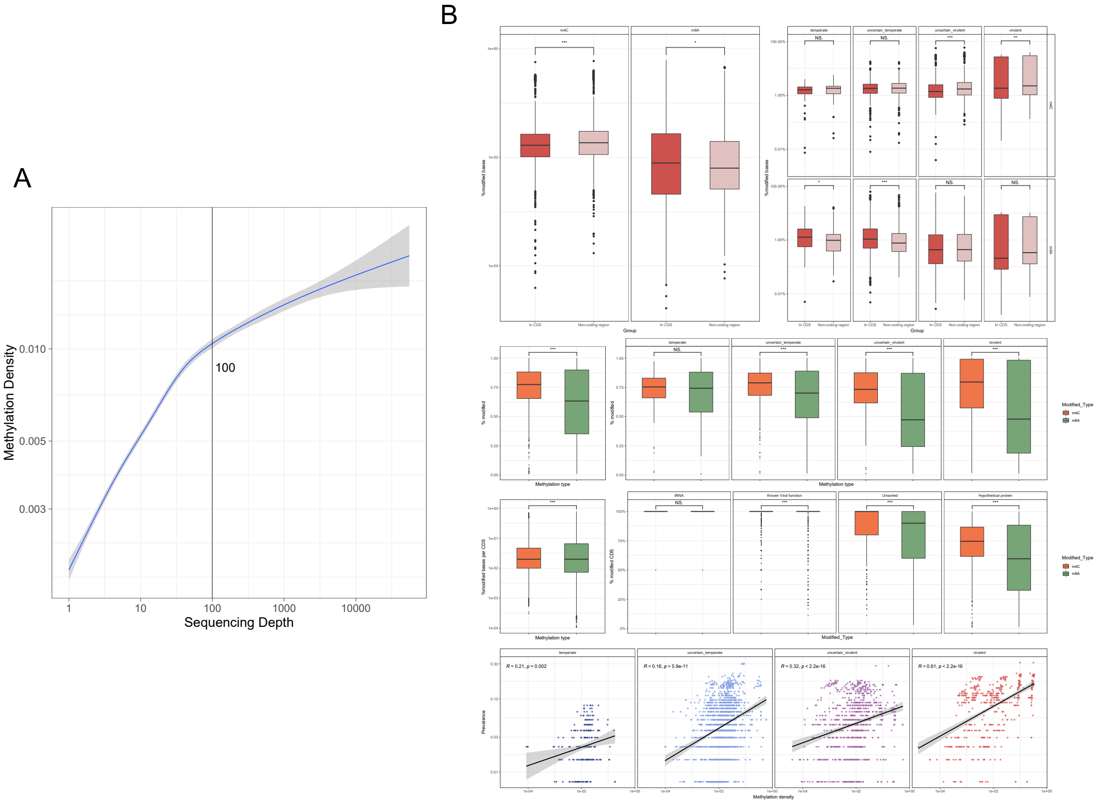

Figure S4, A) The rarefaction of phage methylation density with increasing TGS coverage. The lines represent the coverage under 100. The methylation density among CDS and non-CDS region, methylation density among different gene functions, and phage fitness under the coverage cutoff of **B)** 100. Limiting our analysis to phages with long-reads coverages did not affect our result.

Figure S5

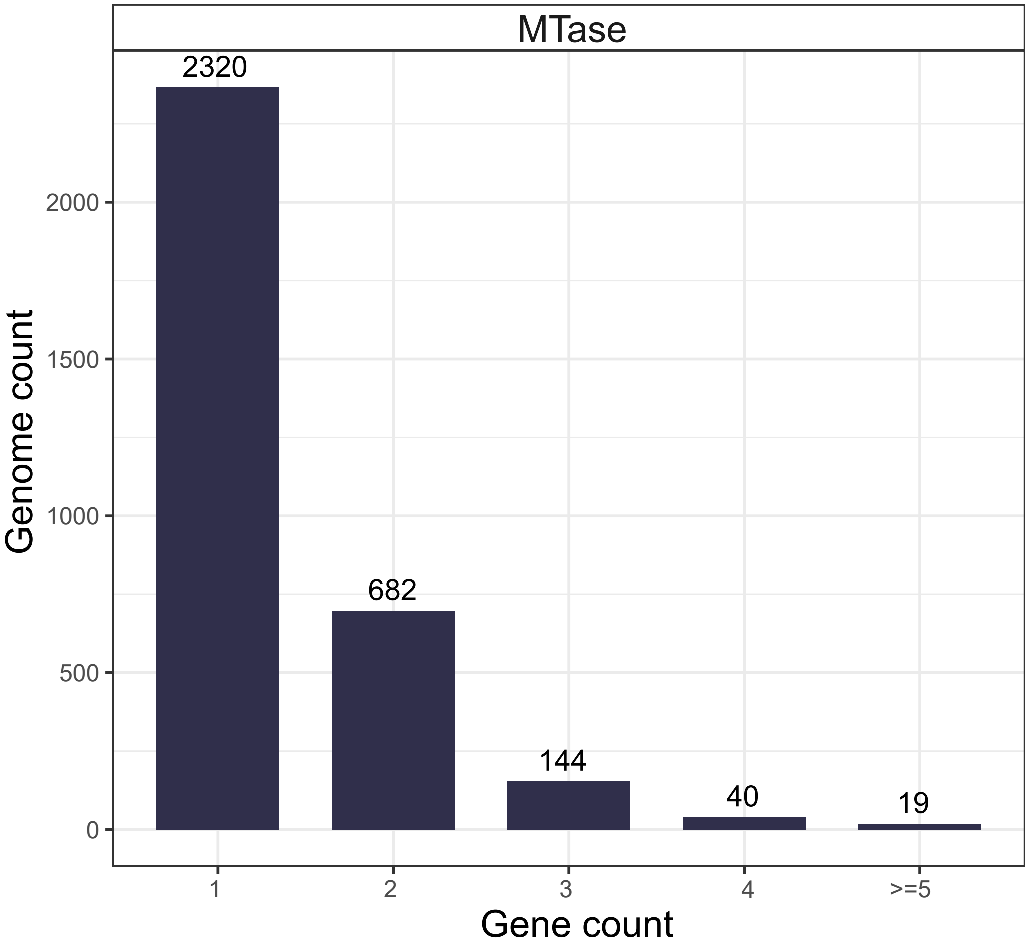

Figure S5, The number of genomes encode different count of MTases. Most phages contain one MTase gene, but some can encode multiple ones.

Figure S6

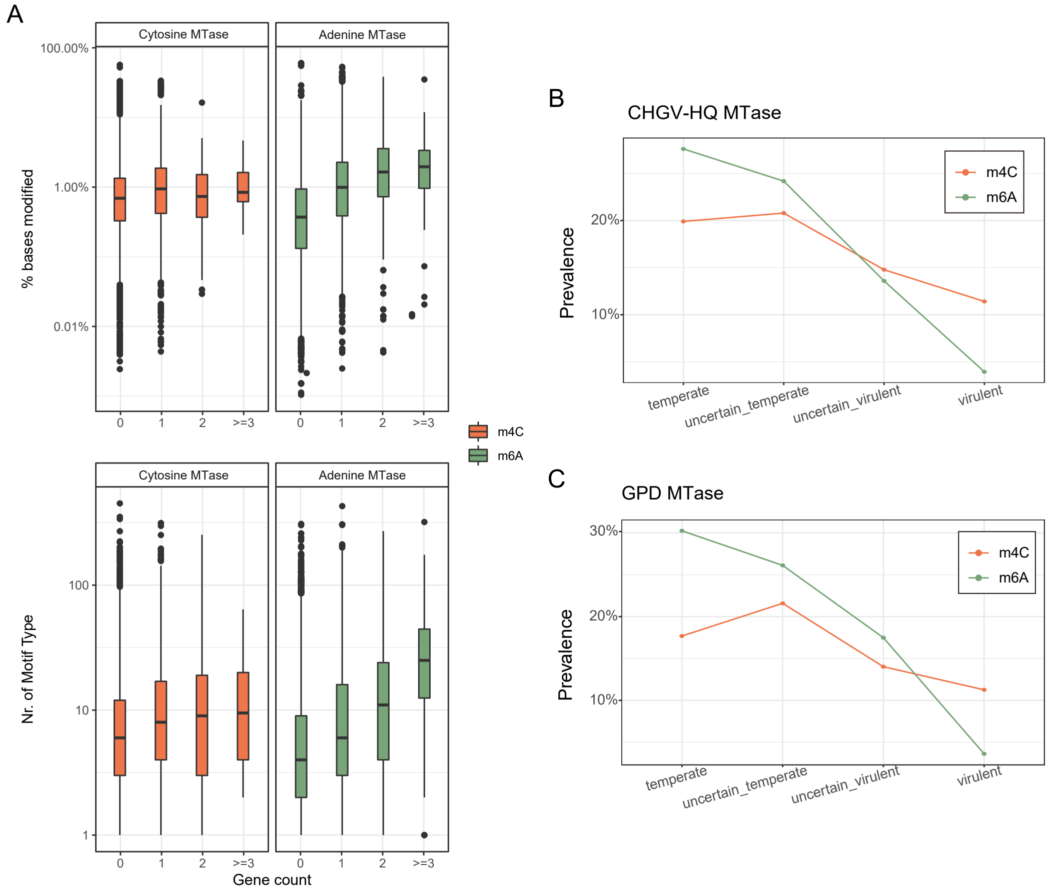

Figure S6, A) Both the methylation densities and the numbers of methylation motifs increased with increasing number of phage-encoded MTases responsible for individual modification types. A higher prevalence of MTase genes from CHGV **B)** and GPD **C)** is associated with decreasing phage virulence. The trends in the individual MTase types, i.e., MTases responsible for m4C and m6A modifications were largely the same.

Figure S7

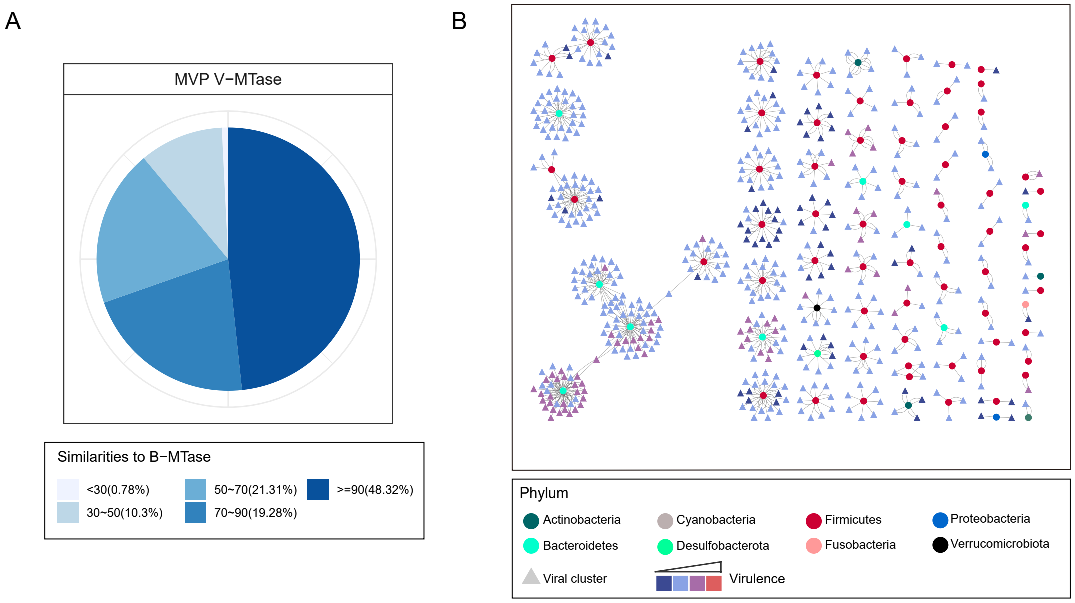

Figure S7, A) 48.32% of the MVP V-MTases share over 90% protein sequence identity with their bacteria-encoded homologs. Further prove that most the gut phage MTases are of bacterial origin. **B)** The phage-host interaction network-based predictions from MTase genes.

Figure S8

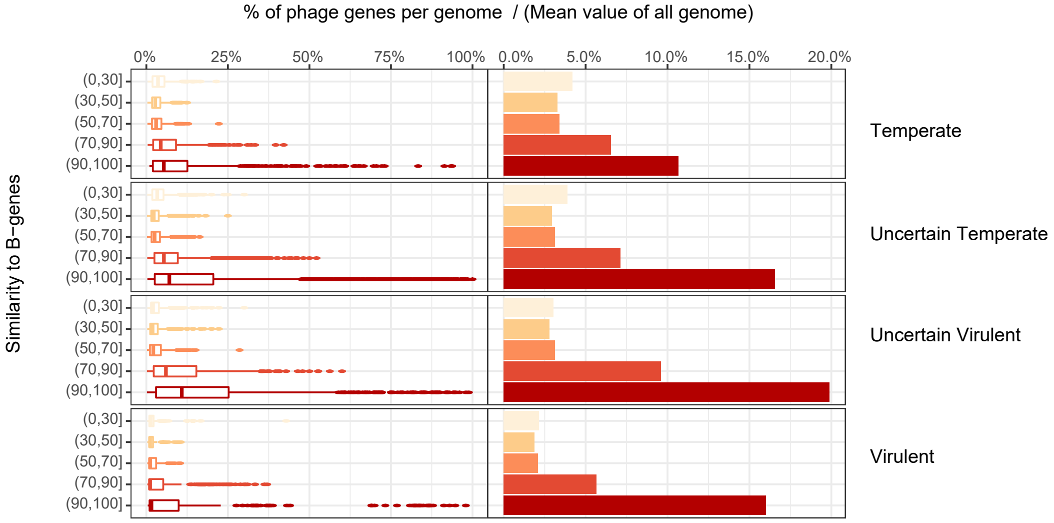

Figure S8 20% genes per phage genome (V-genes) share significant protein similarities with the UHGG2 gut bacteria(B-genes). The trends stay the same among different lifestyles.

Figure S9

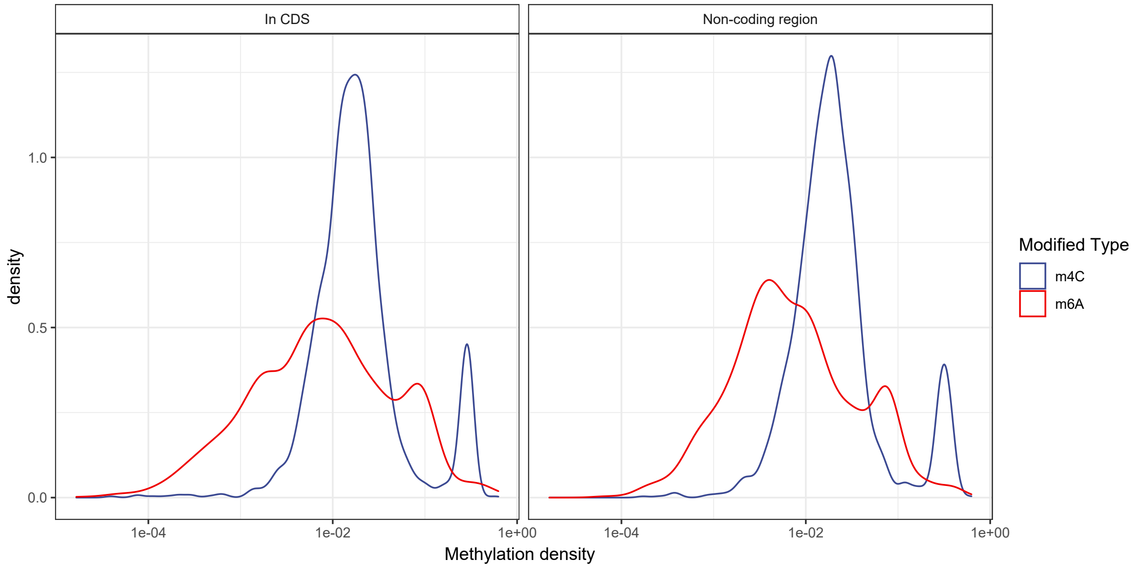

Figure S9 Most methylation-positive genomes are with high methylation density, no matter coding and non-coding regions.
